# Author Correction: Electron energy increase in a laser wakefield accelerator using up-ramp plasma density profiles

**DOI:** 10.1038/s41598-020-75483-x

**Published:** 2020-10-21

**Authors:** Constantin Aniculaesei, Vishwa Bandhu Pathak, Hyung Taek Kim, Kyung Hwan Oh, Byung Ju Yoo, Enrico Brunetti, Yong Ha Jang, Calin Ioan Hojbota, Jung Hun Shin, Jong Ho Jeon, Seongha Cho, Myung Hoon Cho, Jae Hee Sung, Seong Ku Lee, Björn Manuel Hegelich, Chang Hee Nam

**Affiliations:** 1 Center for Relativistic Laser Science, Institute for Basic Science (IBS), Gwangju, 61005 Republic of Korea; 2 Advanced Photonics Research Institute, Gwangju Institute of Science and Technology (GIST), Gwangju, 61005 Republic of Korea; 3 Department of Physics and Photon Science, GIST, Gwangju, 61005 Republic of Korea; 4 Scottish Universities Physics Alliance, University of Strathclyde, Department of Physics, Glasgow, G4 0NG United Kingdom

Correction to: *Scientific Reports* https://doi.org/10.1038/s41598-019-47677-5, published online 02 August 2019

This Article contains errors. In the Introduction,

“Plasma frequency is defined by the formula ${\omega }_{p}=$($4\pi {n}_{0}{e}^{2}$/${m}^{0.5}$ with ${n}_{0}$ being plasma density, $m$ is electron rest mass and $e$ is the elementary charge.”

should read:

“Plasma frequency is defined by the formula ${\omega }_{p}= \left(4\pi {n}_{0}{e}^{2} / {m}^{0.5}\right)$ with ${n}_{0}$ being plasma density, $m$ is electron rest mass and $e$ is the elementary charge.”

Additionally, in the Discussion,

“In the PIC simulations, we applied 3 types of density profiles, shown in Fig. 2a: one trapezoidal profile with peak density 1 × 10^19^ electrons/cm^3^ (case *i*), one trapezoidal profile with peak density 1.4 × 10^19^ electrons/cm+ (case *iii*) and one profiles with 3 ramps (case *ii*).”

should read:

“In the PIC simulations, we applied 3 types of density profiles, shown in Fig. 2a: one trapezoidal profile with peak density 1 × 10^19^ electrons/cm^3^ (case *i*), one trapezoidal profile with peak density 1.4 × 10^19^ electrons/cm^3^ (case *iii*) and one profiles with 3 ramps (case *ii*).”

Additionally, in the Figure Legend for Figure 2

“Quasi-3D PIC simulation to show the of density ramp on the LWFA: Figure (a) highlights the initial density profiles used to simulate three cases, and (b) compares the electron energy spectrum for the three cases when the maximum energy is obtained at time *t* = 1452 ${\upomega }_{p}^{-1}$ for case (i) and (ii), and at time *t* = 1313 ${\upomega }_{p}^{-1}$ for case (iii). Figure (c–e) show the longitudinal phase space and electric field lineout at the axis (blue line) for the three cases at time *t* = 1194 ${\upomega }_{p}^{-1}$.”

should read:

“Quasi-3D PIC simulation to show the effect of density ramp on the LWFA: Figure (a) highlights the initial density profiles used to simulate three cases, and (b) compares the electron energy spectrum for the three cases when the maximum energy is obtained at time *t* = 1452 ${\upomega }_{p}^{-1}$ for case (i) and (ii), and at time *t* = 1313 ${\upomega }_{p}^{-1}$ for case (iii). Figure (c–e) show the longitudinal phase space and electric field lineout at the axis (blue line) for the three cases at time *t* = 1194 ${\upomega }_{p}^{-1}$.”

Finally, in the Methods,

“Here the results are discussed for three density profiles plotted in Fig. 2(a), where in case (i) The plasma density rises to n_0_ in plasma scale length 900c\ω_p_, stay flat for 800c\ω_p_, and drops down to 0 in next 900c\ω_p_ plasma length. In case (ii) after reaching the density n_0_ in plasma scale length 900c\ω_p_, the electron density further grows to 1.4 n_0_ in next 800c\ω_p_, and in case (iii) the density profile is similar to the case i, with flat density at 1.4 n_0_.”

should read:

“Here the results are discussed for three density profiles plotted in Fig. 2(a), where in case (i) The plasma density rises to n_0_ in plasma scale length 900 c/ω_p_, stays flat for 800 c/ω_p_, and drops down to 0 in next 900 c/ω_p_ plasma length. In case (ii) after reaching the density n_0_ in plasma scale length 900 c/ω_p_, the electron density further grows to 1.4 n_0_ in next 800 c/ω_p_, and in case (iii) the density profile is similar to the case i, with flat density at 1.4 n_0_.”

