# Supplementary material for: Electron energy increase in a laser wakefield accelerator using up-ramp plasma density profiles
Source: Sci Rep. 2019 Aug 2;9:11249. doi: 10.1038/s41598-019-47677-5 (PMC6677811; doi:10.1038/s41598-019-47677-5)
Supplement: Supplementary file 1 — Supplementary for “Electron energy increase in a laser wakefield accelerator using up-ramp plasma density profiles” [file 41598_2019_47677_MOESM1_ESM.docx]

# Supplementary for “Electron energy increase in a laser wakefield accelerator using up-ramp plasma density profiles”

Constantin Aniculaesei^1,#^, Vishwa Bandhu Pathak^1^, Hyung Taek Kim^1,2,*^, Kyung Hwan Oh^1^, Byung Ju Yoo^1^, Enrico Brunetti^4^, Yong Ha Jang^1^, Calin Ioan Hojbota^1,3^, Jung Hun Shin^1^, Jong Ho Jeon^1^, Seongha Cho^1^, Myung Hoon Cho^1^, Jae Hee Sung^1,2^, Seong Ku Lee^1,2^, Björn Manuel Hegelich^1,3^ and Chang Hee Nam^1,3^

^1^Center for Relativistic Laser Science, Institute for Basic Science (IBS), Gwangju 61005, Republic of Korea.

^2^Advanced Photonics Research Institute, Gwangju Institute of Science and Technology (GIST), Gwangju 61005, Republic of Korea

^3^Department of Physics and Photon Science, GIST, Gwangju 61005, Republic of Korea

^4^Scottish Universities Physics Alliance, University of Strathclyde, Department of Physics, Glasgow, G4 0NG, United Kingdom

[*htkim@gist.ac.kr](mailto:*htkim@gist.ac.kr)

[#ca182@ibs.re.kr](mailto:#ca182@ibs.re.kr)

**Experimental results with a straight nozzle**

The gas nozzle was set in straight position (gas flow direction perpendicular to the laser axis) and the laser focal plane fixed longitudinally at 1.5 mm from the centre of the nozzle. Helium gas was used. Various gas density profiles, as seen by the laser, were generated by changing the inlet pressure and the interaction point relative to the exit of the nozzle (the height).

The gas density profiles are shown in Fig. 1a, each colour corresponding to a dataset recorded in the same experimental conditions. The results from each dataset are averaged. The specific experimental conditions for each dataset are summarized in Table 1. The results for the mean electron energy shown in Fig. 1.b present a very small variation, around 5% from 174.8 ± 1.33 MeV (yellow data set in Fig. 1b) to 183.7 ± 5.97 MeV (blue dataset in Fig. 1b) even though the gas density has been varied more than 50% from 0.88×10^19^ atoms/cm^3^ (black curve P1) to 1.37×10^19^ atoms/cm^3^ (blue curve P4). The error bar represents the standard error of the mean. The mean divergence remains quasi-constant, ~58 mrad along the horizontal axis and ~35 mrad along the vertical axis. The stability of the electron beam energy is the only one that suffers changes, especially at higher gas densities where, for example, it shows a 4.4 times increase of the mean energy standard deviation between dataset obtained with the profile P3 compared with the dataset obtained the profile P4.

Fig. 1 The experimental results obtained with the straight nozzle. In Fig.1a are shown the gas density profiles and in Fig.1b are shown the corresponding mean energy for each dataset. The peak density has been adjusted more than 50%, from 0.88×10^19^ atoms/cm^3^ (black curve) to 1.37×10^19^ atoms/cm^3^ (blue curve) and the mean electron energy showed a negligible variation of 5%. The divergence remains quasi-constant, around 58 mrad along the horizontal axis and around 35 mrad along the vertical axis. The laser propagates from right to left.

| Profile | No of  shots | Height  (mm) | Inlet  pressure  (bar) | Peak density  (×10^19^ atoms/cm^3^) |
| --- | --- | --- | --- | --- |
| 1 (Black) | 12 | 3 | 10 | 0.88 |
| 2 (Red) | 13 | 4 | 15 | 1.18 |
| 3 (Green) | 12 | 5 | 15 | 1.06 |
| 4 (Blue) | 14 | 5 | 20 | 1.37 |

Table 1 contains the experimental conditions for the straight nozzle case.

In Table 2 the first ramp is the ramp that interacts first with the laser (the laser propagates from right to left in Fig. 1c). The 1st ramp starts where the gas density is 1 × 1018 atoms/cm3 and stops at the beginning of the second ramp. The 2nd ramp stops where the gas density reaches its maximum and the 3rd ramp starts at the end of 2nd ramp end ends where the gas density drops to 1 × 1018 atoms/cm3.

**Experimental conditions for the tilted nozzle case**

Fig. 2 shows the gas density profile corresponding to each dataset with the laser propagating from the right side towards the left side. The density profiles were obtained by tilting the nozzle, adjusting the interaction point in the vertical direction and changing the inlet pressure, matching the parameters in Table 2.

| Tilt angle α  (^o^) | No of  shots | Height  (mm) | Inlet  Pressure  (bar) | Peak density  (×10^19^ atoms/cm^3^) | 1^st^  ramp length  (mm) | 2^nd^  ramp length  (mm) | 3^rd^  ramp length  (mm) | Electron mean  peak energy  (MeV) |
| --- | --- | --- | --- | --- | --- | --- | --- | --- |
| 30 (navy) | 20 | 4 | 15 | 1.09 | 2.184 | 0.869 | 1.744 | 239 |
| 20 (dark yellow) | 23 | 3 | 10 | 0.79 | 1.741 | 0.942 | 1.492 | 243 |
| 10 (yellow) | 21 | 3 | 15 | 1.13 | 2.016 | 0.801 | 1.946 | 262 |
| 5 (magenta) | 15 | 5 | 20 | 1.17 | 2.9 | 0 | 2.9 | 226 |
| 0 (cyan) | 12 | 5 | 15 | 1.06 | 2.029 | 0.806 | 2.029 | 175 |

Table 2 The experimental conditions for the tilted nozzle case
